# Profiling and bioinformatic analysis of circular RNA expression regulated by c-Myc

**DOI:** 10.18632/oncotarget.17788

**Published:** 2017-05-10

**Authors:** Qiheng Gou, Ke Wu, Jian-Kang Zhou, Yuxin Xie, Lunxu Liu, Yong Peng

**Affiliations:** ^1^ Department of Thoracic Surgery and Lab of Non-Coding RNAs in Diseases, State Key Laboratory of Biotherapy, West China Hospital, Sichuan University, and Collaborative Innovation Center for Biotherapy, Chengdu, Sichuan 610041, China; ^2^ Cancer Center, West China Hospital, Sichuan University, Chengdu, Sichuan 610041, China

**Keywords:** circular RNA, microRNA, c-Myc, profiling, bioinformatics analysis

## Abstract

The c-Myc transcription factor is involved in cell proliferation, cell cycle and apoptosis by activating or repressing transcription of multiple genes. Circular RNAs (circRNAs) are widely expressed non-coding RNAs participating in the regulation of gene expression. Using a high-throughput microarray assay, we showed that Myc regulates the expression of certain circRNAs. A total of 309 up- and 252 down-regulated circRNAs were identified. Among them, randomly selected 8 circRNAs were confirmed by real-time PCR. Subsequently, Myc-binding sites were found to generally exist in the promoter regions of differentially expressed circRNAs. Based on miRNA sponge mechanism, we constructed circRNAs/miRNAs network regulated by Myc, suggesting that circRNAs may widely regulate protein expression through miRNA sponge mechanism. Lastly, we took advantage of Gene Ontology and KEGG analyses to point out that Myc-regulated circRNAs could impact cell proliferation through affecting Ras signaling pathway and pathways in cancer. Our study for the first time demonstrated that Myc transcription factor regulates the expression of circRNAs, adding a novel component of the Myc tumorigenic program and opening a window to investigate the function of certain circRNAs in tumorigenesis.

## INTRODUCTION

Circular RNAs (circRNAs) were recently discovered as a novel type of endogenous noncoding RNA and emerged in the central field of RNA research. Unlike linear RNA with 5′ caps and 3′ tails, circRNAs form covalently closed loop structures, which enable higher stability and resistance against RNA exonuclease [1, 2]. Along with deep sequencing and bioinformatics, researchers recognized that many circRNAs are abundant and conserved across species [3, 4].

Originally, circRNAs were considered as a byproduct of RNA splicing and lack functional significance [5]. However, recent studies revealed that some circRNAs can regulate alternative splicing and modulate gene expression via microRNA sponge [6]. For example, ciRS-7 (also named CDR1as) contains over 60 miR-7 microRNA response elements (MREs) and forms the RNA-induced silencing complex with Argonaute (AGO) protein. Consequently, it strongly suppresses miR-7 activity, promoting the expression of miR-7 target genes [6, 7]. Moreover, hundreds of circRNAs are regulated during epithelial–mesenchymal transition (EMT), indicating that certain circRNAs may affect cell migration, invasion and metastasis [8]. Currently, the knowledge about circRNA is just the tip of the iceberg. Therefore, the function and regulation of circRNAs require further investigation.

Myc is a transcription factor encoded by the proto-oncogene *c-Myc*, and its expression is tightly regulated during development. However, Myc expression is commonly elevated in a variety of cancers through different mechanisms, such as gene translocation, gene amplification, transcriptional activation and enhanced protein stability [9]. Early reports indicated that Myc plays a pivotal role in cell proliferation, differentiation and apoptosis by activating or repressing transcription of multiple genes, including protein-coding genes and non-coding RNA genes [10]. For example, in cooperation with its heterodimerization partner Max, Myc directly binds to E-box element with the consensus sequence 5′-CACGTG-3′ and activates transcription of *PRDX3* gene, encoding a mitochondrial protein of the peroxiredoxin gene family, participating in mitochondrial homeostasis and neoplastic transformation [11]. Myc was reported to repress CDKN2B (p15) expression by binding Miz-1 and displacing Miz-1 cofactors to silence *CDKN2B* gene expression in the absence of TGF-β [12, 13].

MicroRNA (miRNA) is a family of small non-coding RNAs that regulate many biological processes [14], and their expression are temporally and spatially regulated by Myc transcription factor. O'Donnell et al. reported that Myc directly activates the expression of miR-17-92 miRNA cluster, leading to modulate E2F1 expression to control cell proliferation. Using high-throughput gene chips, the expression of many miRNAs was found to be down-regulated by Myc through direct binding to promoters or conserved regions upstream of such miRNAs. Therefore, regulation of miRNA expression by Myc is a fundamental component of the Myc tumorigenic program. Besides miRNA, Myc also regulates the expression of long non-coding RNAs (lncRNAs). Kim et al. identified several Myc-regulated lncRNAs (named MYCLos) in colorectal cancer to modulate the expression of CDKN1A (p21) and CDKN2B (p15), suggesting a novel regulatory mechanism of Myc-repressed target genes through lncRNAs [15]. Subsequently, more lncRNAs such as lncRNA-MIF and MINCR were found to be activated by Myc and affect cell cycle and glycolysis [16, 17].

To date, whether Myc regulates circRNA expression remains unclear. In this study, we took advantage of high-throughput microarray to identify Myc-regulated circRNAs in human B-cell line, P493-6, that harbors a tetracycline-repressible Myc transgene. We also analyzed the Myc-binding sites upstream of circRNAs to deduce how Myc regulates circRNAs expression. On the basis of circRNAs/miRNAs sponge and MREs mechanisms, we established a network including Myc-regulated circRNAs, interacting miRNAs, and their target mRNAs, elucidating a novel mechanism of Myc-mediated function through circRNAs.

## RESULTS

### Identification of Myc-regulated circRNAs

**Figure 1:**
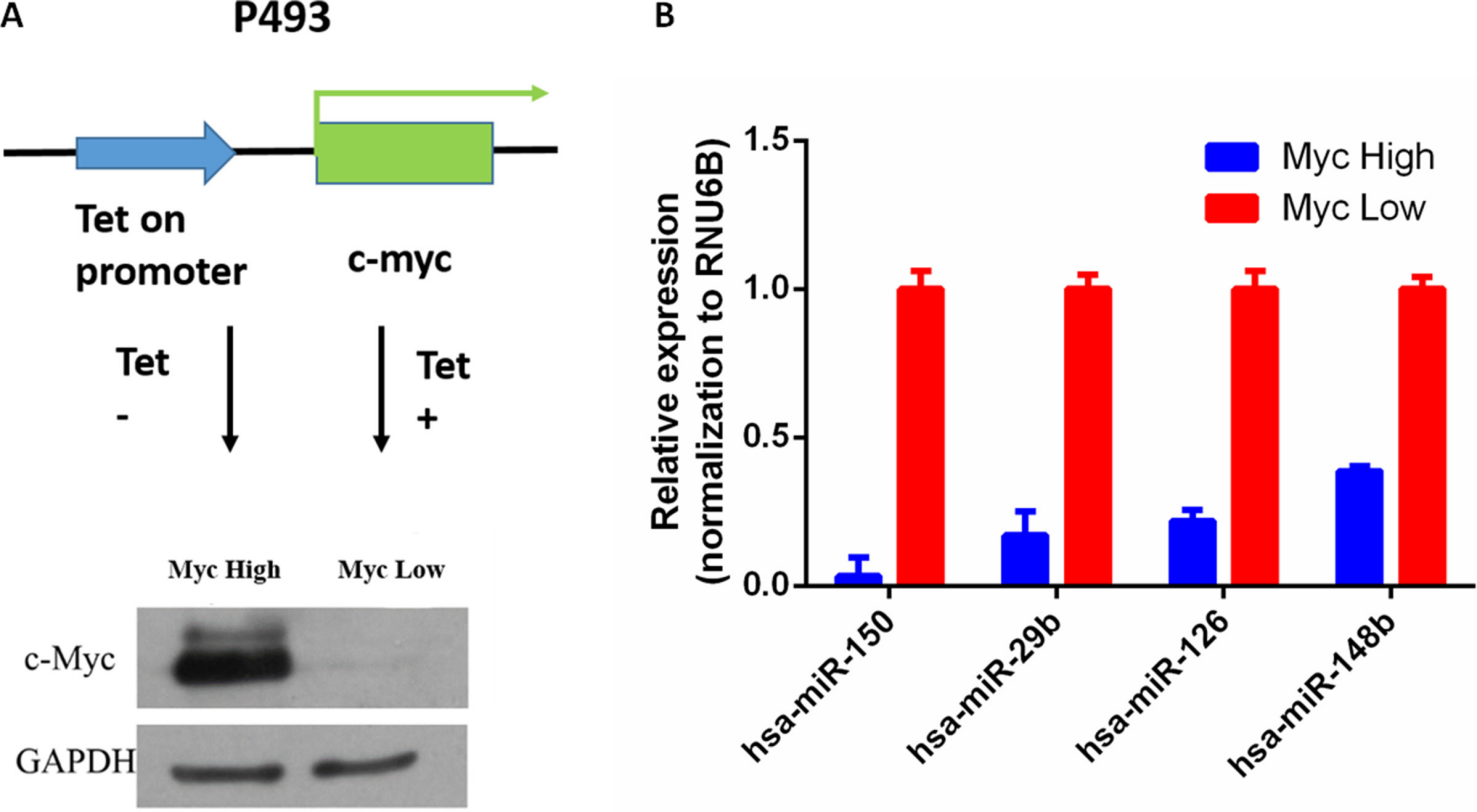
Regulation of miRNA expression by Myc in P493-6 cells (**A**) Myc expression by Western blot in P493-6 cells with or without tetracycline. (**B**) miRNA expression by qRT-PCR in P493-6 cells; RUN6B served as normalization, *p* < 0.05.

In order to identify Myc-regulated circRNA, we used the P493-6 cells, which are Epstein-Barr virus–immortalized human B cells carrying a tetracycline (tet)-repressible allele of Myc [18]. These cells are tumorigenic in immunocompromised mice and represent a model of human B cell lymphoma. In these cells, treatment with tetracycline for 72 h strongly down-regulated the expression of Myc mRNA and protein levels (Figure 1A). To validate the functional effects of Myc expression in these cells, we measured miRNA expression by real-time PCR. Consistent with previous reports, expression of several well-known miRNAs, including miR-150, miR-29b, miR-126 and miR-148b, were significantly down-regulated after Myc induction [19] (Figure 1B). Therefore, the RNA samples should have the expected Myc-dependent gene expression profile.

**Figure 2:**
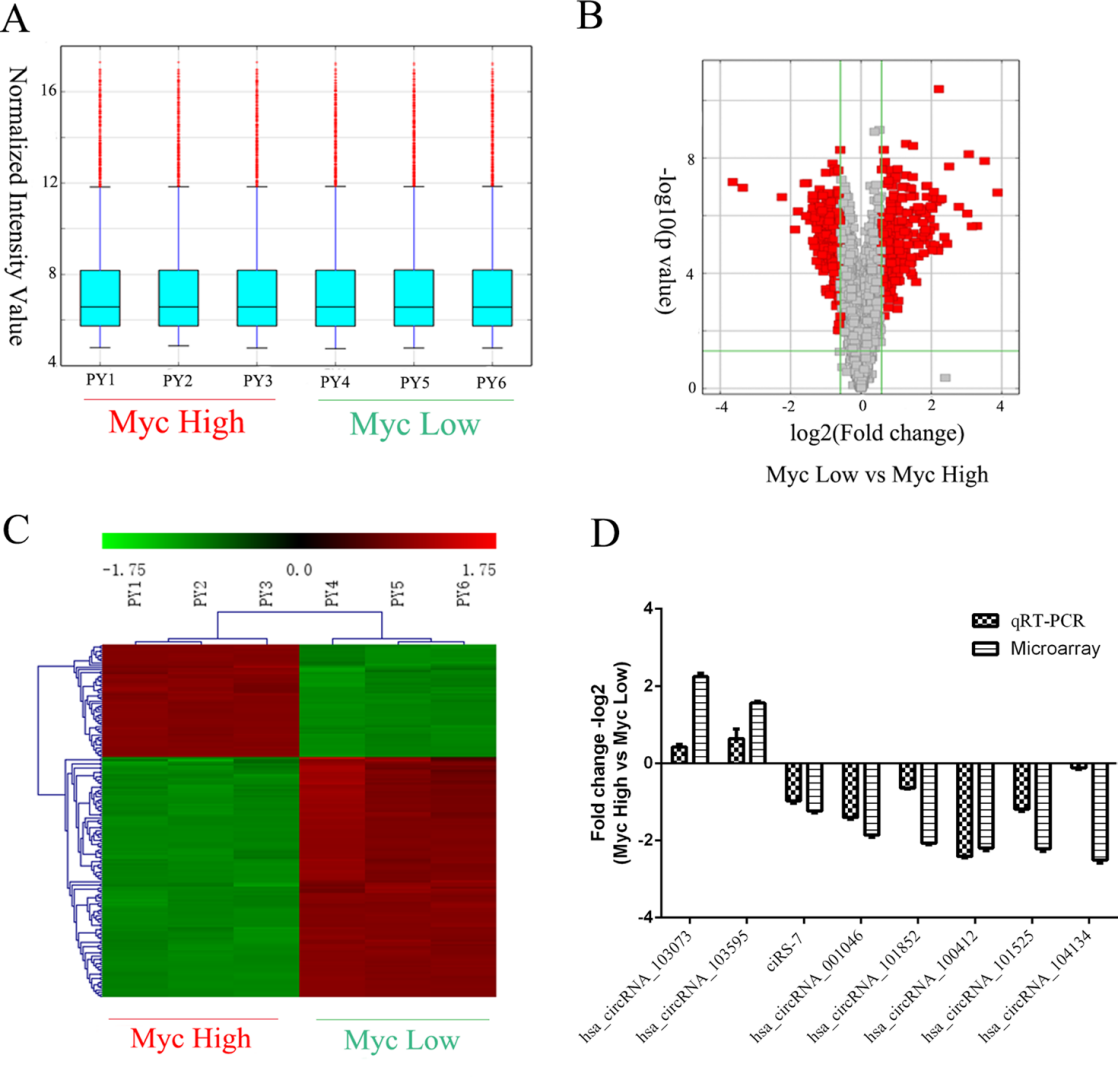
Screening and validation of circRNAs regulated by Myc (**A**) Box plot showing the homogeneous distributions of the intensities among high Myc (PY1-3) and low Myc (PY4-6) samples. (**B**) Volcano plots showing visualization of differential expression of circRNAs regulated by Myc. The vertical lines correspond to 2.0-fold (log2 scaled) up- or down-regulation, respectively. The horizontal line represents a *p*-value of 0.05 (−log10 scaled). The red points in the plot represent the differentially expressed circRNAs with statistical significance. (**C**) Heat map and hierarchical clustering showing differential expression of circRNAs by Myc. Each group has three biological replicates. (**D**) Validation of differentially expressed circRNAs by qRT-PCR and its comparison with microarray data. The vertical axis shows the mean of fold change (−log2 transformed) of each circRNA as measured by qRT-PCR and microarray analysis, respectively.

Using high-throughput microarray assay, we profiled circRNA expression of P493-6 cells with the high-Myc (−tet) and low-Myc (+tet) states. A total of 4517 circRNAs were detected in two groups of samples, each group with three independent repeats. From the box-plot analysis, we visualized the homogeneous distribution of the intensities in all samples (Figure 2A). Subsequently, we statistically compared two groups of profile differences by *t*-test, and circRNAs with fold change ≥1.5, *p*-value ≤ 0.05 and false discovery rate (FDR) < 0.05 were selected as the significantly differentially expressed. The volcano plot and hierarchical cluttering showed the differential expression of circRNAs between high-Myc and low-Myc groups (Figure 2B and 2C). Totally, there were 561 circRNAs with differential expression, among which 309 circRNAs were up-regulated while 252 were down-regulated by the Myc transcription factor.

The top 20 up- and down-regulated circRNAs are listed in Table 1. To validate microarray data, we measured the expression of randomly selected 8 circRNAs by real-time PCR Supplementary Table 1. As expected, hsa_circRNAs_103073 and hsa_circRNAs_103595 were significantly up-regulated by Myc, while other six circRNAs (hsa_circRNAs_001046, hsa_circRNAs_101852, hsa_circRNAs_100412, hsa_circRNAs_101525, hsa_circRNAs_104134 and ciRS-7) were down-regulated by Myc. Therefore, our microarray data, consistent with qRT-PCR results, were reliable (Figure 2D, Supplementary Table 1).

**Table 1:** The top 20 up-regulated and down-regulated circRNAs ranked by fold changes in microarray data

circRNA	P-value	FDR	Fold change	circRNA type	chrom	circRNA	P-value	FDR	Fold change	circRNA type	chrom
Down-regulated circRNAs						Up-regulated circRNAs					
hsa_circRNA_103557	1.57E-07	1.47E-05	14.7167552	exonic	chr3	hsa_circRNA_101181	6.75E-08	1.13E-05	12.5254635	exonic	chr12
hsa_circRNA_103497	1.26E-08	6.08E-06	11.4269497	exonic	chr3	hsa_circRNA_101180	1.07E-07	1.24E-05	10.2866641	exonic	chr12
hsa_circRNA_001493	2.27E-06	4.23E-05	9.8219918	antisense	chr18	hsa_circRNA_103073	2.25E-07	1.47E-05	4.7539949	exonic	chr20
hsa_circRNA_104308	2.34E-06	4.26E-05	8.8998602	exonic	chr7	hsa_circRNA_101469	3E-06	4.99E-05	3.6729	exonic	chr15
hsa_circRNA_102993	7.31E-09	4.13E-06	8.3578094	exonic	chr20	hsa_circRNA_102187	7.02E-07	2.44E-05	3.4591693	exonic	chr17
hsa_circRNA_104203	8.4E-07	2.57E-05	8.0936767	exonic	chr6	hsa_circRNA_000993	7.53E-08	1.17E-05	3.0249577	intragenic	chr9
hsa_circRNA_104315	4.93E-07	2.14E-05	6.8741003	exonic	chr7	hsa_circRNA_103595	1.02E-06	2.82E-05	2.951369	exonic	chr4
hsa_circRNA_104134	1.96E-08	7.06E-06	5.6746289	exonic	chr6	hsa_circRNA_000911	7.35E-08	1.17E-05	2.9036055	intronic	chr21
hsa_circRNA_103972	9.39E-06	0.000103	5.4419796	exonic	chr5	hsa_circRNA_102402	1.49E-06	3.39E-05	2.726367	exonic	chr19
hsa_circRNA_400019	5.42E-06	7.33E-05	5.1660866	intronic	chr11	hsa_circRNA_101706	8.94E-07	2.67E-05	2.613709	exonic	chr16
hsa_circRNA_101853	2.58E-07	1.57E-05	4.9006312	exonic	chr16	hsa_circRNA_102847	2.64E-07	1.57E-05	2.5965107	exonic	chr2
hsa_circRNA_100946	2.78E-07	1.63E-05	4.7008926	exonic	chr11	hsa_circRNA_102396	5.42E-07	2.26E-05	2.5826961	exonic	chr19
hsa_circRNA_101525	4E-11	1.81E-07	4.6328319	exonic	chr15	hsa_circRNA_104168	1.94E-07	1.47E-05	2.5788327	exonic	chr6
hsa_circRNA_100412	1.29E-05	0.000124	4.5727993	exonic	chr1	hsa_circRNA_400027	7.41E-06	8.7E-05	2.5687314	intronic	chr15
hsa_circRNA_101531	1.65E-05	0.000143	4.5571984	exonic	chr15	hsa_circRNA_100352	7.81E-07	2.54E-05	2.502524	exonic	chr1
hsa_circRNA_101055	3.31E-07	1.78E-05	4.4270141	exonic	chr12	hsa_circRNA_104828	1.14E-05	0.000114	2.4992246	exonic	chr9
hsa_circRNA_100844	1.03E-05	0.000107	4.2446029	exonic	chr11	hsa_circRNA_000881	3.35E-07	1.78E-05	2.4922513	intronic	chr17
hsa_circRNA_101852	2.44E-06	4.33E-05	4.2009477	exonic	chr16	hsa_circRNA_104852	1.18E-06	3.08E-05	2.4698025	exonic	chr9
hsa_circRNA_103541	1.49E-07	1.46E-05	4.1995852	exonic	chr3	hsa_circRNA_100141	1.68E-06	3.57E-05	2.4125369	exonic	chr1
hsa_circRNA_101353	2.42E-07	1.56E-05	4.1540307	exonic	chr14	hsa_circRNA_104101	3.72E-05	0.00024	2.3939667	exonic	chr6

Note: CircRNA ID: The circRNA ID is in circBase (http://circbase.mdc-berlin.de). CircRNA type: The circRNAs are classified into 5 types: “exonic”, “intronic”, “antisense”, “intragenic” and “intergenic”. P-value: P-value calculated from paired t-test. FDR: FDR is calculated from Benjamini Hochberg FDR. Fold Change: The absolute ratio (no log scale) of normalized intensities between two conditions.

### Prediction of Myc-binding sites in the circRNA promoter

**Figure 3:**
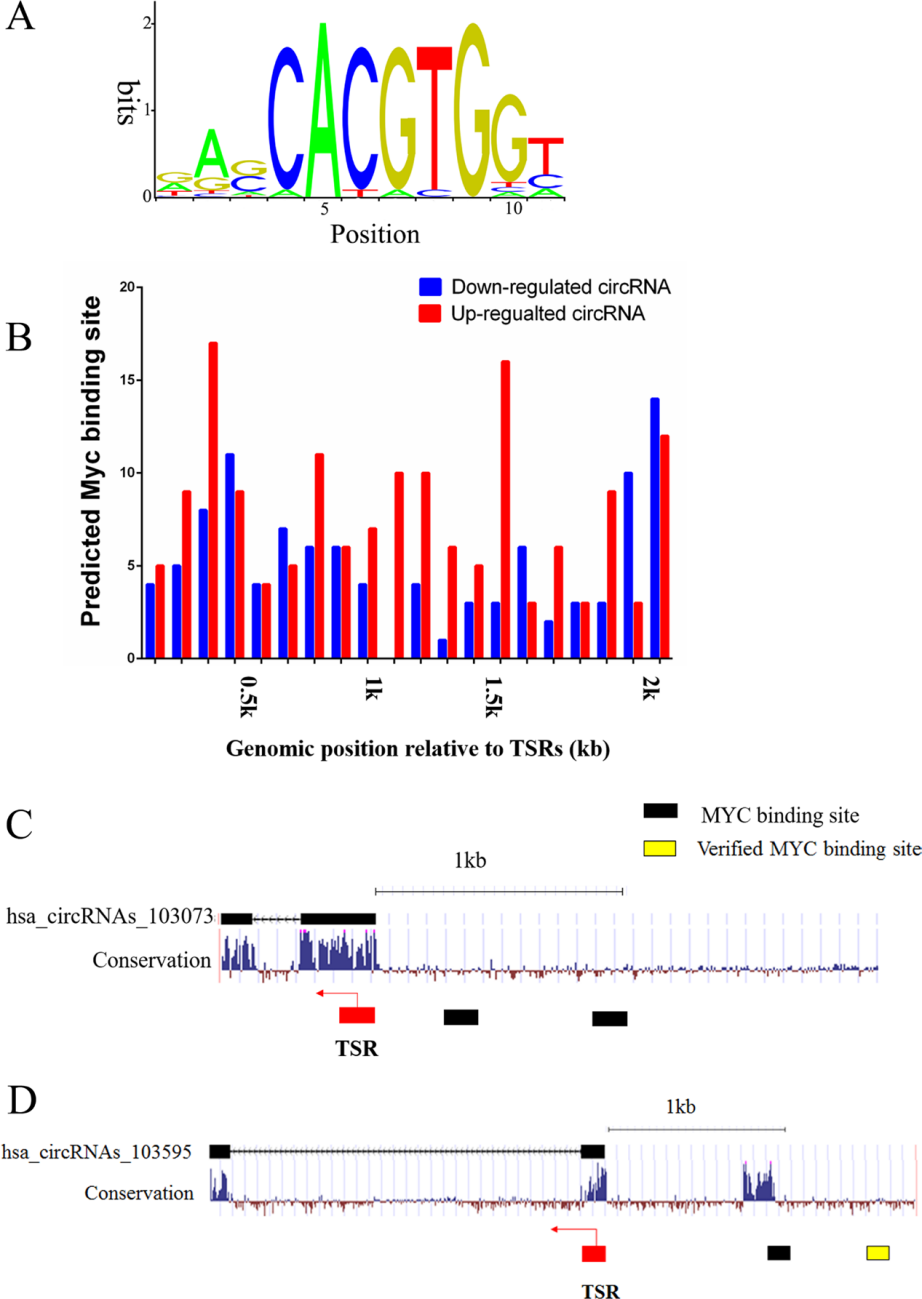
Prediction of Myc-binding sites in the promoter regions of differentially expressed circRNAs (**A**) The conserved Myc-binding site sequence. (**B**) Myc-binding sites spreading over the 2 kb regions from TSR of up- and down-regulated circRNAs, the right diagram represents a significant difference, *p* < 0.05. (**C**) Predicted Myc-binding sites in the promoter region upstream of hsa_circRNAs_103073. (**D)** Predicted and validated Myc-binding sites in the promoter region upstream of hsa_circRNAs_103595. Arrows denote the transcription direction, black squares denote the predicted Myc-binding sites and yellow square denotes the verified Myc-binding site.

In accordance with Myc's regulatory transcription mechanisms, we hypothesized that Myc regulates expression of circRNAs by directly interacting with their promoters. Intriguingly, bioinformatic analysis showed that the E-box consensus sequence CACGTG was found in more than a half circRNAs (220 up- and 101 down-regulated) (Figure 3A). Because many studies demonstrate that Myc occupies the 2kb promoter regions of protein-coding genes [20, 21], so we analyzed the similar regions surrounding the transcription start region (TSR) of circRNAs using the motif-based sequence analysis tool FIMO, a MEME Suite (4.11.1) component, and found that the Myc-binding sites widely exist in the candidate circRNAs (Figure 3B). For example, both hsa_circRNAs_103073 (Alias: hsa_circ_0060558) and hsa_circRNAs_103595 (Alias: hsa_circ_0069086) have the Myc-binding sites near TSR (Figure 3C and 3D). Furthermore, our predicted Myc-binding site of hsa_circRNAs_103595 was verified by ChIP-seq from ENCODE (http://genome.ucsc.edu/), supporting that the Myc-binding sties we predicted are reliable. Therefore, we deduced that Myc regulates circRNA expression via binding to the E-box elements. Moreover, we verified the promoter region of PLTP (Homo sapiens phospholipid transfer protein), the parental gene of hsa_circRNAs_103073, by ChIP-seq from ENCODE (http://genome.ucsc.edu/). As a result, we identified conserved Myc-binding sites located at upstream of the PLTP promoter. The results revealed that Myc possibly participated in the activation of circRNAs via activation of their parental gene.

### Prediction of circRNA-miRNA-mRNA associations

**Figure 4:**
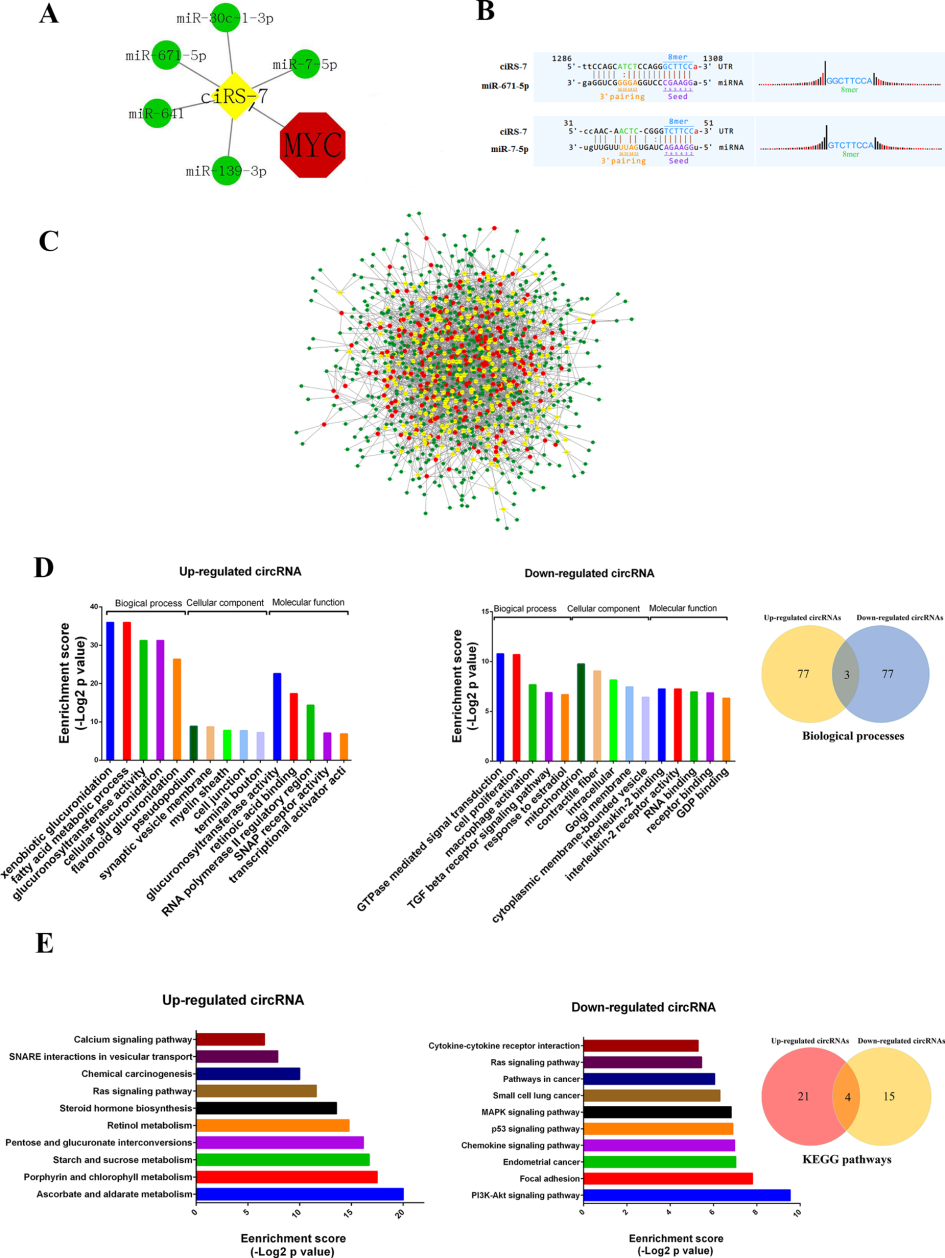
The circRNA/miRNA network, GO and KEGG analysis (**A**) Interaction between Myc-repressed cisR-7 and miRNAs. (**B**) The representative MREs of cisR-7 combine with miR-671 and miR-7. (**C**) The network of circRNAs/miRNAs consisting of 309 up-regulated circRNAs (red nodes), 252 down-regulated circRNAs (yellow nodes), and their target miRNAs (green nodes) by fold change > 1.5, *p* < 0.05, and FDR < 0.05. (**D**) The top 5 Gene ontology processes of up- and down-regulated circRNAs, Venn diagram indicates the overlapped biological processes in both up- and down-regulated circRNAs. (**E**) KEGG pathway analysis showed the top 10 pathways of up- and down-regulated circRNAs, Venn diagram indicates the overlapped pathways in up- and down-regulated circRNAs.

circRNAs were reported to function as miRNA ‘sponge’ to sequester and competitively suppress miRNA activity [6], so we analyzed interactions between circRNAs and their target miRNAs using Arraystar's miRNA target prediction software based on TargetScan and miRanda. The 309 up-regulated and 252 down-regulated circRNAs were theoretically predicted by conserved seed-matching sequence. As a result, a total of 664 miRNAs could be combined with the differentially expressed circRNAs (Supplementary Table 2). For example, ciRS-7, a Myc-repressed circRNA, was predicted to interact with 5 miRNAs (miR-7-5p, miR-671-5p, miR-641, miR-30c-1-3p and miR-139-3p) (Figure 4A and 4B). The entire network of circRNA/miRNA interaction was delineated using Cytoscape v3.40 (Figure 4C), suggesting that circRNAs may widely regulate protein expression through miRNA sponge mechanism.

To deduce the function of circRNAs, Gene Ontology (GO) enrichment analysis was performed according to the enriched mRNAs targeted by circRNA-combined miRNAs. Figure 4D showed the top 5 GO processes of each subgroup (BP, CC and MF) for the up- and down-regulated circRNAs. Interestingly, regulation of cell proliferation, GTPase activity and transcription from RNA polymerase II promoter exists in both up- and down-regulated circRNAs (Figure 4D and Supplementary Table 3), consistent with the fact that Myc as a key factor controlling the cell proliferation and gene expression.

KEGG pathway enrichment analysis was also performed to comprehend pathways and molecular interactions in which the differentially expressed circRNAs could be involved (Supplementary Table 4). Figure 4E revealed the top 10 pathways related to up- and down-regulated circRNAs. Notably, the Ras signaling pathway and pathways in cancer were the top pathways for both up- and down-regulated circRNAs, implying that Myc-related circRNAs may significantly affect such pathways to participate in tumorigenesis.

## DISCUSSION

In recent years, emerging studies have revealed that circRNAs act as a novel non-coding RNA to be involved in various diseases, especially in cancers [5]. Utilizing the high-throughput RNA sequencing and bioinformatics, the increasing amount of conservative circRNAs was discovered [22]. However, regulation of circRNAs expression and their function still remain unclear. Myc is a crucial transcription factor to regulate the expression of protein coding genes and non-coding RNA genes such as miRNAs and lncRNAs [23, 24]. But there is no report about whether Myc regulates circRNA expression. Thus, we took advantage of circRNA microarray to explore Myc-regulated circRNAs.

In this study, we profiled circRNA expression in P493-6 B cells with high or low Myc expression and found that 309 circRNAs were up-regulated while 252 were down-regulated via Myc expression. Among them, randomly selected 8 circRNAs (hsa_circRNAs_103073, hsa_circRNAs_103595, ciRS-7, hsa_circRNAs_001046, hsa_circRNAs_101852, hsa_circRNAs_100412, hsa_circRNAs_101525, and hsa_circRNAs_104134) were further confirmed by qRT-PCR, validating that our microarray data are reliable. To the best of our knowledge, this is the first time to demonstrate that expression of circRNAs is regulated by Myc transcription factor. Because Myc plays a pivotal role in tumorigenesis through affecting cell proliferation and apoptosis, so our study open a window to investigate circRNA function.

A large body of evidence indicated that Myc could activate or repress transcription of multiple genes. For instance, Myc binds to the E-box element with consensus sequence CACGTG and actives transcription of the cyclin-dependent kinase 4 (CDK4), thus promoting cell-cycle progress via CDK4 [11]. Myc also directly activates the expression of miR-17-92 cluster via binding to the canonical E-box in the promoter, modulating E2F1 expression to control cell proliferation[10, 25, 26]. Moreover, Myc promotes the expression of long noncoding RNA MYCLo-1 to modulate the expression of CDKN1A (p21) and CDKN2B (p15) genes [15]. On the contrary, Myc represses the expression of many genes. Some researchers demonstrated that the Myc is coupled with Miz-1 to reduce the expression of certain genes, such as p15INK4b [27]. Brenner et al. found that Myc and Dnmt3a form a ternary complex with Miz-1 to repress the p21Cip1 promoter [28]. Myc represses expression of miR-15, miR-29, miR-126, miR-148b and miR-150 through direct binding to promoters or conserved regions upstream of such miRNAs [19, 29]. Thus, it would be interesting to investigate whether the E-box sequence exists in the promoter of Myc-regulated circRNAs. Here we analyzed the 2kb promoter regions of circRNAs using the motif-based sequence analysis tool FIMO, a MEME Suite component, and found that the Myc-binding sites widely exist in the promoters of these circRNAs. Therefore, Myc may regulate expression of certain circRNAs via directly binding to the promoter's conserved E-box sequences.

circRNAs could function as miRNA ‘sponge’ to regulate gene expression, so we analyzed interactions between circRNAs and their target miRNAs using Arraystar's miRNA target prediction software, and found that 664 miRNAs could be combined with the Myc-regulated circRNAs. Moreover, we constructed the entire network of circRNA/miRNA interaction by Cytoscape software. These data suggest that circRNAs may widely regulate gene expression through miRNA sponge mechanism.

Notably, the ciRS-7 was significantly down-regulated by Myc according to our microarray and qRT-PCR data. Using the bioinformatic analysis, we predicted that ciRS-7 could interact with the following 5 miRNAs: hsa-miR-7-5p, hsa-miR-671-5p, hsa-miR-641, hsa-miR-30c-1-3p and hsa-miR-139-3p. Hansen et al. reported that ciRS-7 contains more than 60 conserved miR-7 target sites, and it is highly and widely associated with Argonaute (AGO) proteins in a miR-7-dependent manner, thus regulating expression of miR-7 targets [6, 7, 30]. Moreover, miR-671 was found to repress ciRS-7 expression in an Ago2-slicer-dependent manner through binding with ciRS-7 [6, 30, 31]. Given the fact that previous literatures are consistent with our prediction of the ciRS-7/miRNA interaction, the entire network of circRNA/miRNA interaction we constructed should be reliable to understand the role of circRNAs on miRNA activity. Besides, we predicted that miR-9 is complementary to hsa_circRNAs_101525, which was abundant in cutaneous squamous cell carcinoma and radioresistant esophageal cancer [32, 33].

To deduce the function of circRNAs, we performed GO enrichment analysis according to the enriched mRNAs targeted by circRNA-combined miRNAs, and found that regulation of cell proliferation, GTPase activity and transcription from RNA polymerase II promoter exists in both up- and down-regulated circRNAs, which is consistent with the important function of Myc in the cell proliferation and gene expression. Such Go enrichment analyses imply that Myc-regulated circRNAs could participate in regulating such biological processes.

Furthermore, KEGG pathway enrichment analysis revealed the pathways and molecular interactions in which the differentially expressed circRNAs could be involved. Among them, the Ras signaling pathway and pathways in cancer were the top pathways for both up- and down-regulated circRNAs. The results suggest that Myc-related circRNAs may significantly affect such pathways to participate in cellular proliferation and tumorigenesis [24, 34, 35].

Based on the literatures, we outlined the mechanism associated with Myc, circRNAs, miRNAs and their targets. The antisense coding ciRS-7 and sense-coding CDR1 in the nucleus were exported to the cytoplasm where the mRNA can be translated. In the presence of miR-671-Ago2-RISC in the nucleus, both ciRS-7 and CDR1 mRNA were degraded simultaneously [30]. Furthermore, we hypothesize that Myc negatively controls the ciRS-7 transcription via binding to the promoter region. In the cytoplasm, ciRS-7 could block miR-7 via more than 60 MREs and increase the expression of miR-7 target genes in turn, such as EGFR, RAF1, PAK1, mTOR, and other genes involved in cell proliferation and metastasis [36–38]. Because miR-671 is nearly perfectly complementary to ciRS-7 [6, 30, 39], so the function of ciRS-7 is under control of miR-671 in the cytoplasm [40]. Even if the circularization could protect it from nuclease cleavage, it is not sufficient to counteract Ago2 slicer activity in RISC (Supplementary Figure 1).

In conclusion, our study for the first time revealed the circRNA expression signatures under the Myc regulation. Moreover, Myc-binding sites were found to generally exist in the promoter regions of differentially expressed circRNAs. Based on miRNA sponge mechanism, we constructed circRNAs/miRNAs network regulated by Myc transcription factor. Lastly, we took advantage of GO and KEGG analyses to point out that Myc-regulated circRNAs could play an important role in cell proliferation through affecting Ras signaling pathway and pathways in cancer. Currently, we are investigating the function and mechanism of circRNAs regulated by Myc.

## MATERIALS AND METHODS

### Cell culture

P493-6 B cells were cultured in RPMI 1640 with 100 U/ml penicillin, 100 mg/ml streptomycin, and 10% fetal bovine serum at 37°C in a humidified incubator containing 5% of CO_2_. For repression of Myc, P493-6 cells were cultured in media with 100 ng/ml tetracycline hydrochloride for 72 h.

### Western blot analysis

Total cell proteins were separated on polyacrylamide gels and transferred onto PVDF membrane. Then the membrane was blocked for 1 h at room temperature prior to incubation with polyclonal rabbit anti-Myc antibody (Abcam, ab32072) or anti-β-actin antibody (Abcam, ab8227) at 4°C overnight. After washed with TBST, membrane was incubated in horseradish peroxidase-conjugated anti-rabbit antibody for 1 h, and developed by SuperSignal West Pico Chemiluminescent Substrate (Thermo Scientific).

### RNA isolation, RNA labeling and RNA hybridization

Cell lines were subjected to standard TRIzol RNA isolation (Life Technologies, Carlsbad, CA, USA). The concentrations of the RNA samples were determined by means of OD260 using a NanoDrop ND-1000 instrument. The integrity of RNA was assessed by electrophoresis in a denaturing agarose gel. Sample labeling and array hybridization were performed according to the manufacturer's protocol (Arraystar Inc.). Briefly, total RNAs were digested with Rnase R (Epicentre, Inc.) to remove linear RNAs and enrich circular RNAs. Then, the enriched circular RNAs were amplified and transcribed into fluorescent cRNA utilizing a random priming method (Arraystar Super RNA Labeling Kit; Arraystar). The labeled cRNAs were purified by RNeasy Mini Kit (Qiagen). The concentration and specific activity of the labeled cRNAs (pmol Cy3/μg cRNA) were measured by NanoDrop ND-1000. 1 μg of each labeled cRNA was fragmented by adding 5 μl 10 × Blocking Agent and 1 μl of 25 × Fragmentation Buffer, then heated the mixture at 60°C for 30 min, finally 25 μl 2 × Hybridization buffer was added to dilute the labeled cRNA. 50 μl of hybridization solution was dispensed into the gasket slide and assembled to the circRNA expression microarray slide. The slides were incubated for 17 hours at 65°C in an Agilent Hybridization Oven. The hybridized arrays were washed, fixed and scanned using the Agilent Scanner G2505C.

### Real-time PCR validation

After RNA extraction, M-MLV reverse transcriptase (Invitrogen, Carlsbad, CA) was used for synthesizing cDNA according to manufacturer's instructions. The expression level of the circRNAs was evaluated by qPCR using the SYBR Green assay. Specific divergent primers were designed to amplify the circular transcripts. PCR was performed in a 10-μl reaction volume, including 2 μl of cDNA, 5 μl 2 × Master Mix, 0.5 μl of Forward Primer (10 μM), 0.5 μl of Reverse Primer (10 μM) and 2 μl of double-distilled water. The reaction was set to 95°C for 10 min for predenaturation, then at 95°C for 10 s and at 60°C for 60 s repeated for 40 cycles. RNU6B served as a reference. Both target and reference were amplified in triplicate wells, and the relative level of each circRNA was calculated using the 2^−ΔΔCt^ method.

### Prediction of Myc binding sites

To confirm direct Myc regulation of the differentially expressed circRNAs, −2 kb sequence from the transcription start site of circRNAs parental gene were assessed the presence of Myc-binding sites by means of Motif-based sequence analysis tools, MEME Suite (4.11.1) component, FIMO.

### Annotation for circRNA/miRNA interaction, GO analysis, and KEGG analysis

Putative targets of circRNAs were predicted by means of miRBase (http://www.mirbase.org/index.shtml). Putative targets of microRNAs were predicted by Targetscan (http://www.targetscan.org/vert_71/). GO analysis was performed in terms of biological processes, cellular components and molecular functions, which were identified by Database from Annotation, Visualization and Integrated Discovery (DAVID;http://www.david.abcc.ncifcrf.gov/). Biological pathways defined by Kyoto Encyclopedia of Genes and Genomes (KEGG; http://www.genome.jp/kegg/) were also identified by Database from Annotation, Visualization and Integrated Discovery (DAVID; http://www.david.abcc.ncifcrf.gov/).

### Statistical analysis

The results were reported as mean ± SD for triplicate measurements. Statistically significant differences between groups were estimated by Student's *t* test using the SPSS software (ver. 13.0). Differences with *p* < 0.05 were considered statistically significant. All the analysis using DAVID involved the Fisher Exact test. When this test yielded *p* < 0.05, the relevant data were considered statistically significant.

## SUPPLEMENTARY MATERIALS FIGURES AND TABLES







## Abbreviations

circRNAs: Circular RNAs
ceRNA: competing endogenous RNA
MREs: microRNA response elements
GO analysis: Gene Ontology analysis
AGO: Argonaute
FDR: false discovery rate
ChIP: chromatin immunoprecipitation.
