# Supplementary material for: Profiling and bioinformatic analysis of circular RNA expression regulated by c-Myc
Source: Oncotarget. 2017 May 10;8(42):71587–96. doi: 10.18632/oncotarget.17788 (PMC5641074; doi:10.18632/oncotarget.17788)
Supplement: Supplementary file 1 [file oncotarget-08-71587-s001.pdf]

Profiling and bioinformatic analysis of circular RNA expression regulated by c-Myc

Supplementary Materials

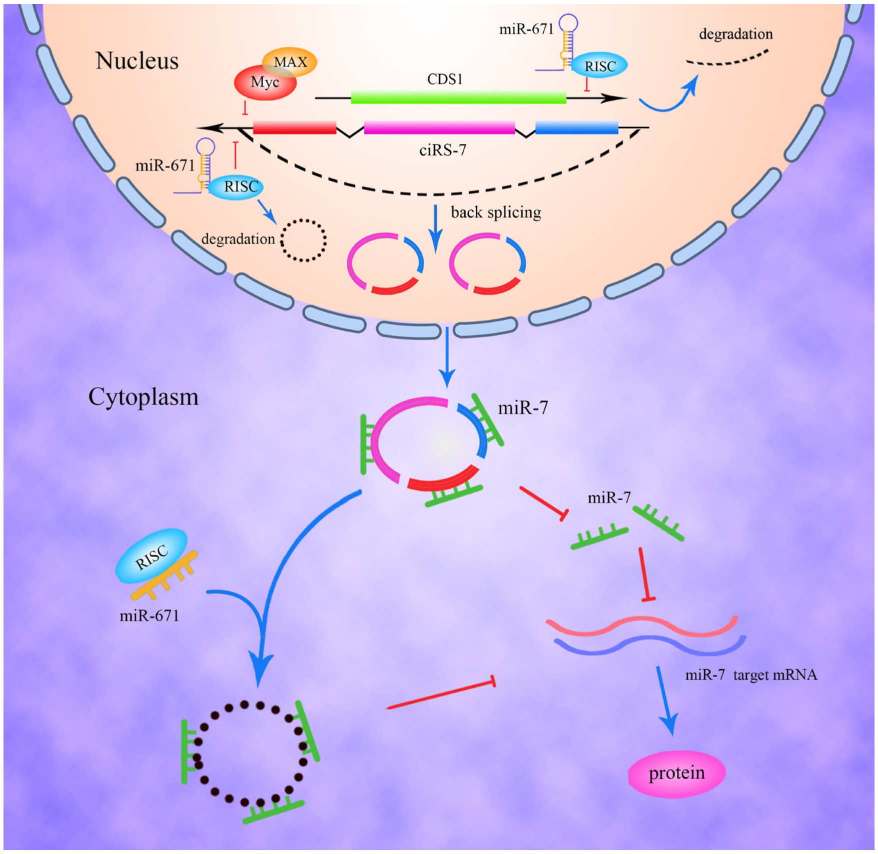

Supplementary Figure 1: The interaction of ciRS-7 with Myc and miRNAs. The Myc-MAX complex may repress the ciRS-7 transcription by its binding to the promoter region. In the nucleus, miR-671-Ago2-RISC complex destabilizes ciRS-7 and CDR1 mRNA. The ciRS-7 is exported to the cytoplasm where it interacts with miR-7 through over 60 MREs, thus increasing the expression of miR-7 target genes. Under the control of miR-671, ciRS-7 is degraded and miR-7 is released from ciRS-7-miR-7 sponge to decrease its target gene expression.

Supplementary Table 1: Primers used in qRT-PCR

Gene ID	primer sequence	TM(°C)	Product length(bp)
β-actin (H)	F:5′ GTGGCCGAGGACTTTGATTG3′<br>R :5′ CCTGTAACAACGCATCTCATATT3′	60	73
5S rRNA	F:5′ GGCCATACCACCCTGAA3′<br>R:5′ GGCGGTCTCCCATCCAA3′	60	89
hsa_circRNA_101181	F:5′ AACAAGACCCTGCTCACCTCA3′<br>R:5′ GAAGGAACAGAGCCTGGGTAGA3′	60	179
hsa_circRNA_103073	F:5′ CATGACTATGGACGCCCGTC3′<br>R:5′ TGGTGCCAGAGGGCTTGAT3′	60	163
hsa_circRNA_103595	F:5′ CAGGACATTTTGCCTCGGTCT3′<br>R:5′ GGTGCGTTGCGTACATGTCTC3′	60	189
hsa_circRNA_100412	F:5′ CACGAATATGCCTTGGCTGTT3′<br>R:5′ ACATCCTGTCACCAAAGCGTAA3′	60	163
hsa_circRNA_101852	F:5′ TGGACCAGGATACTTGTTCA3′<br>R:5′ GTCCTCACTGCCACTAAAGC3′	60	174
hsa_circRNA_105055	F:5′ GTCTTCCATCAACTGGCTCAATA3′<br>R:5′ GGATTGTCTGGAAGATGTGGAT3′	60	226
hsa_circRNA_103557	F:5′ CTGCTATGGGACTATTGCTGT3′<br>R:5′ GGTATATGACAATGGTTCTCCAC3′	60	127
hsa_circRNA_104134	F:5′ ATAGAGGATGGCAAGGAAGACT3′<br>R:5′ TGTGCTCAGGGATGGTAGAAA3′	60	113
hsa_circRNA_101525	F:5′ CTTTATTCATGGCTGCTATCT3′<br>R:5′ GTGTTTGTAAGGGTTCTGGTA3′	60	208
hsa_circRNA_001046	F:5′ CAACCTCCTTAGGCATGTCTGT3′<br>R:5′ GAAAATAGGACTTGAGATGGGAAC3′	60	142

Supplementary Table 2: Differentially expressed circRNAs and their target miRNAs. See Supplementary_Table_2

Supplementary Table 3: The GO terms of Biogical Process analysis of upregulated and downregulated circRNAs. See Supplementary_Table_3

Supplementary Table 4: The KEGG analysis of upregulated and downregulated circRNAs

Overlapped KEGG pathways

Term	Feature
hsa04014	Ras signaling pathway
hsa04144	Endocytosis
hsa04060	Cytokine-cytokine receptor interaction
hsa05200	Pathways in cancer

The KEGG pathways associated with the upregulated circRNAs

Category	Term	Feature	*P*Value	Fold Enrichment
KEGG_PATHWAY	hsa04014	Ras signaling pathway	3.19E-04	2.609602966
KEGG_PATHWAY	hsa04144	Endocytosis	0.036423199	1.811704701
KEGG_PATHWAY	hsa04060	Cytokine-cytokine receptor interaction	0.06394684	1.762126756
KEGG_PATHWAY	hsa05200	Pathways in cancer	0.07948153	1.500687711
KEGG_PATHWAY	hsa00053	Ascorbate and aldarate metabolism	9.36E-07	10.74480249
KEGG_PATHWAY	hsa00860	Porphyrin and chlorophyll metabolism	5.41E-06	7.390604891
KEGG_PATHWAY	hsa00500	Starch and sucrose metabolism	9.17E-06	6.097249035
KEGG_PATHWAY	hsa00040	Pentose and glucuronate interconversions	1.37E-05	7.760135135
KEGG_PATHWAY	hsa00830	Retinol metabolism	3.58E-05	5.253014553
KEGG_PATHWAY	hsa00140	Steroid hormone biosynthesis	8.26E-05	5.351817335
KEGG_PATHWAY	hsa05204	Chemical carcinogenesis	9.77E-04	3.880067568
KEGG_PATHWAY	hsa04130	SNARE interactions in vesicular transport	0.004221648	5.477742448
KEGG_PATHWAY	hsa04713	Circadian entrainment	0.010933608	2.940682788
KEGG_PATHWAY	hsa04020	Calcium signaling pathway	0.010333964	2.305868726
KEGG_PATHWAY	hsa04360	Axon guidance	0.020512866	2.44413705
KEGG_PATHWAY	hsa05032	Morphine addiction	0.026361838	2.728838729
KEGG_PATHWAY	hsa04725	Cholinergic synapse	0.025772827	2.516800584
KEGG_PATHWAY	hsa04721	Synaptic vesicle cycle	0.050724284	2.956241956
KEGG_PATHWAY	hsa05202	Transcriptional misregulation in cancer	0.04327418	2.032416345
KEGG_PATHWAY	hsa04911	Insulin secretion	0.054663812	2.556279809
KEGG_PATHWAY	hsa04726	Serotonergic synapse	0.057941319	2.299299299
KEGG_PATHWAY	hsa00310	Lysine degradation	0.084476244	2.98466736
KEGG_PATHWAY	hsa04750	Inflammatory mediator regulation of TRP channels	0.090701645	2.240039008
KEGG_PATHWAY	hsa04723	Retrograde endocannabinoid signaling	0.090701645	2.240039008
KEGG_PATHWAY	hsa04728	Dopaminergic synapse	0.099695292	2.018896946

The KEGG pathways associated with the downregulated circRNAs

Category	Term	Feature	*P*Value	Fold Enrichment
KEGG_PATHWAY	hsa05213	Endometrial cancer	0.00762527	3.453269857
KEGG_PATHWAY	hsa04115	p53 signaling pathway	0.008405717	3.060852828
KEGG_PATHWAY	hsa05219	Bladder cancer	0.099884216	2.805781759
KEGG_PATHWAY	hsa05222	Small cell lung cancer	0.012658513	2.640735773
KEGG_PATHWAY	hsa05211	Renal cell carcinoma	0.067494822	2.4172889
KEGG_PATHWAY	hsa04917	Prolactin signaling pathway	0.094341917	2.213010965
KEGG_PATHWAY	hsa05412	Arrhythmogenic right ventricular cardiomyopathy (ARVC)	0.094341917	2.213010965
KEGG_PATHWAY	hsa05220	Chronic myeloid leukemia	0.099303127	2.182274701
KEGG_PATHWAY	hsa04510	Focal adhesion	0.004481658	2.07028557
KEGG_PATHWAY	hsa04062	Chemokine signaling pathway	0.007913328	2.062628753
KEGG_PATHWAY	hsa04066	HIF-1 signaling pathway	0.068925198	2.06139068
KEGG_PATHWAY	hsa04151	PI3K-Akt signaling pathway	0.001345977	1.886786574
KEGG_PATHWAY	hsa04068	FoxO signaling pathway	0.068255193	1.870521173
KEGG_PATHWAY	hsa04010	MAPK signaling pathway	0.008873625	1.855792659
KEGG_PATHWAY	hsa04014	Ras signaling pathway	0.022814014	1.787754749
KEGG_PATHWAY	hsa04060	Cytokine-cytokine receptor interaction	0.025509935	1.764334381
KEGG_PATHWAY	hsa05166	HTLV-I infection	0.034079817	1.67246599
KEGG_PATHWAY	hsa05200	Pathways in cancer	0.015116655	1.599224209
KEGG_PATHWAY	hsa04144	Endocytosis	0.064143486	1.57211118
